# From Resilience to Burnout: Psychological Features of Italian General Practitioners During COVID-19 Emergency

**DOI:** 10.3389/fpsyg.2020.567201

**Published:** 2020-10-02

**Authors:** Cinzia Di Monte, Silvia Monaco, Rachele Mariani, Michela Di Trani

**Affiliations:** Department of Dynamic and Clinical Psychology, Sapienza University of Rome, Rome, Italy

**Keywords:** burnout, resilience, general practitioners, pandemic, coping, coronavirus disease 2019

## Abstract

During the COronaVIrus Disease 2019 (COVID-19) pandemic in Italy, general practitioners (GPs) are ensuring continued access to primary care for citizens while also absorbing more of the impact of the crisis than most professional groups. The aim of this study is to explore the relationships between dimensions of burnout and various psychological features among Italian GPs during the COVID-19 emergency. A group of 102 GPs completed self-administered questionnaires available online through Google Forms, including Maslach Burnout Inventory (MBI), Resilience Scale, Intolerance of Uncertainty Scale Short Form (IU), and Coping Inventory for Stressful Situations (CISS). Cluster analysis highlighted four distinct burnout risk profiles: Low Burnout, Medium Risk, High Risk, and High Burnout. The High Burnout group showed both lower Resilience and lower CISS Task-oriented coping strategy than the Medium Risk group and higher IU Prospective than the Low Burnout group. Results of a linear regression analysis confirmed that CISS Emotion-oriented style positively predicted MBI Emotional Exhaustion, CISS Task-oriented and Emotion-oriented emerged as significant predictors (negatively and positively, respectively) of MBI Depersonalization, and Resilience positively predicted MBI Personal Accomplishment. In conclusion, the results showed that the COVID-19 emergency had a significant impact on GPs’ work management. Implementing task-oriented problem management, rather than emotional strategies, appears to protect against burnout in these circumstances. It is possible that the emotions related to the pandemic are too intense to be regulated and used productively to manage the professional issues that the COVID-19 pandemic presents.

## Introduction

When a pandemic strikes, as COronaVIrus Disease 2019 (COVID-19) has over the last half year, the health system and the people working within it must adapt rapidly to cope with new challenges (Kaba and Kitaw, 2020). Healthcare professionals may be forced to put their lives at risk by serving as the first line of defense. This has certainly been the case in Italy, which, as of May 5, 2020, was the second highest in number of COVID-19 infections (211,938) and highest in total number of deaths (29,079) in Europe (European Centre for Disease Prevention and Control, n.d.). Recently the “Federazione nazionale degli ordini dei medici chirurghi e degli odontoiatri FNOMCeO (2020)” website has created a section for all the physicians who have died fighting COVID-19. As of May 5, 2020, there are 154 reported victims, of which 52 are general practitioners (GPs), one of the most affected categories.

The pandemic has an impact on the mental health of the general population through rapid and abrupt changes, producing high levels of stress and depression, especially in those most at risk to contract the virus (Rodríguez-Rey et al., 2020). Under this tremendous existential threat, GPs continue to ensure access to primary care for citizens. In reporting infections, supporting regional assistance networks, treating patients with minor symptoms, and taking care of the worried well, they play a critical role in suppressing any pandemic (Opstelten et al., 2009) and in confronting disaster conditions (Redwood-Campbell and Abrahams, 2011). Ultimately, their primary care work prevents overcrowding in emergency departments (Levi et al., 2019) and consequently limits the spread of the disease.

In this context, GPs must cope with professional and personal challenges, highlighting big differences between countries (Burns et al., 2020). For example, in Italy, GPs have historically played an important and personal role in the lives of families, but in this pandemic situation, GPs modified their practice methods by using telephone calls and other digital approaches (Fiorino et al., 2020). With these changes, some typical functions of primary care, including physical examinations and immunizations, have been unavoidably neglected (Thornton, 2020) while GPs are tasked with new responsibilities, such as additional safety protocols, learning new technology, and daily e-mails for prescriptions.

Thus, now more than ever, Italian GPs are facing abnormal burdens of work, stressful clinical and organizational conditions, and emotional charges that are challenging their ability to resist stress.

Burnout is a psychological syndrome that occurs in response to chronic job-related stress, with features involving emotional exhaustion, depersonalization, and a sense of reduced personal accomplishment (Maslach et al., 1986). It is common among healthcare professionals who are frequently exposed to high levels of occupational stress, especially due to overwhelming emotional and interpersonal interactions (Bria et al., 2012). Burnout among healthcare professionals has been the subject of a great deal of research because at its higher levels, it is associated with negative impacts on individual physicians, patients, and healthcare organizations and systems (West et al., 2018). All of the research on GPs and burnout has been conducted in the context of daily work; the appropriateness of applying conclusions from that work to pandemic situations is questionable.

The majority of Italian studies presented in the literature were focused on physicians working in a hospital setting. Bressi et al. (2008) reported that levels of burnout were high in hemato-oncology physicians with specific demographic profiles and for those experiencing physical exhaustion and working with demanding patients. Sanfilippo et al. (2018) highlighted that cardiac anesthesiologists are at high and moderately high risk of developing burnout syndrome. Mannocci et al. (2019) showed that 40% of 70 healthcare professionals in hematological units had a high level of emotional exhaustion. Another Italian study compared the burnout levels of GPs to those of hospital physicians: GPs had higher levels of emotional exhaustion than hospital physicians but there were no significant differences for other burnout dimensions explored (Grassi and Magnani, 2000). This study showed that GPs have a high risk of developing burnout syndrome. Recent studies examined the prevalence of burnout during the COVID-19 emergency in health professionals working in Northern Italy (Giusti et al., 2020), showing high levels of burnout especially in Emotional Exhaustion and reduced Personal Accomplishment. These burnout findings were significantly higher than those detected in other Italian samples before the COVID-19 outbreak, especially for Emotional Exhaustion (Barello et al., 2020).

Some individual psychological features can contribute to or prevent the development of burnout.

Psychological resilience, described as the ability to “bounce back” from negative emotional experiences and to adopt flexible solutions to the changing demands of stressful experiences (Tugade and Fredrickson, 2004), has emerged as the main protective factor of burnout among nurses (Guo et al., 2018). In a study of 566 surgical residents, Lebares et al. (2017) showed, with statistical significance, that higher levels of resilience were associated with a lower risk of burnout from emotional exhaustion, depersonalization, and low personal accomplishments. Little information is available about GPs’ resilience. In a survey on Australian GPs, Cooke et al. (2013) found an association between high resilience and low burnout.

In addition, the literature has focused on the role of coping strategies in the development or prevention of burnout syndrome. When individuals experience stress, they can rely on coping mechanisms, which can be either problem-focused (actively changing the stressful environment) or emotion-focused (managing the emotional response to the stressor). Endler and Parker (1994) detected three coping styles: task-, emotion-, and avoidance-oriented coping. Other research has demonstrated that task-oriented coping predicts lower burnout among healthcare professionals while emotion-oriented coping predicts increased burnout (Jaracz et al., 2005; Howlett et al., 2015; Rodríguez-Rey et al., 2019).

Finally, another psychological feature related to the ability to regulate stress is the intolerance of uncertainty, defined as “an individual’s dispositional incapacity to endure the aversive response triggered by the perceived absence of salient, key, or sufficient information, and sustained by the associated perception of uncertainty” (Carleton, 2016). In the Cooke et al. (2013) study mentioned above, GPs’ ability to tolerate uncertainty was also explored with greater intolerance being associated high levels of burnout and low resilience.

The majority of the findings discussed in this *Introduction* have involved studies taking place outside of the context of states of emergency, so are not necessarily directly applicable in a pandemic. They are likely of value in establishing a baseline understanding of burnout among medical professionals but clearly it would be useful to examine how the related phenomena function in a pandemic.

The first aim of this study is to explore the relationships between dimensions of burnout and some psychological features (resilience, intolerance of uncertainty, and coping styles) among Italian GPs during the COVID-19 emergency. Its second aim is to identify which psychological and/or demographic features predict higher levels of burnout.

## Materials and Methods

### Participants

The study focused on Italian GPs currently in service in the time period between March 10, 2020, and May 18, 2020, excluding pensioners and other medical specializations. Individuals in training at GP offices and functioning essentially in the same role as GPs, but not yet certified, were included. A total of 102 individuals participated in the study.

### Procedure

We conducted a study on Italian GPs using snowball sampling and self-administered questionnaires. In March 2020, questionnaires were made available online through Google Forms, and several GP Associations and the State Medical Board were involved in data collection that was stopped on May 18, 2020. GPs accepted the informed consent and the privacy policy before the beginning of the questionnaires.

The study was carried out in accordance with the code of ethics of the World Medical Association (Declaration of Helsinki) for experiments involving humans. Ethical approval was granted by the ethics committee of the Department of Dynamic and Clinical Psychology.

### Measures

#### Sociodemographic Questionnaire

The self-administered questionnaire collected data on multiple variables, including years of work experience, age, number of children, etc.

#### Maslach Burnout Inventory

The questionnaire adopted in this study to measure burnout is the Italian validation of the Maslach Burnout Inventory (MBI; Maslach et al., 1986; Sirigatti and Stefanile, 1993), composed of 22 items with a Likert scale from 0 (never) to 6 (daily). It defines burnout in three dimensions: emotional exhaustion (EE), depersonalization (DP), and personal accomplishment (PA). The EE represents the depletion of one’s emotional resources (example: “I feel used up at the end of workday”). The dimension of DP brings a view of coworkers and clients as dehumanized objects instead of people (example: “I feel I treat some patients as if they were impersonal objects”). Finally, the PA reflects feelings of competence, productivity, and successful achievement in one’s work (example: “I feel I’m positively influencing other people’s lives through my work”). For this dimension only, a high score indicates low burnout level. In this study, Cronbach’s alpha was satisfactory for all subscales: EE (*α*: 0.92), DP (*α*: 0.80), PA (*α*: 0.79).

#### Coping Inventory for Stressful Situations

The Coping Inventory for Stressful Situations (CISS; Endler and Parker, 1994) is a questionnaire of 48 items measured on a Likert scale from 1 (not at all) to 5 (very much). It was administered in the Italian validation (Sirigatti and Stefanile, 2009). The questionnaire can bring up three basic dimensions: *task-*, *emotion-*, and *avoidance-oriented coping*. The scale of Task-oriented coping emphasizes an action oriented to the task and on attempts to solve the problem. The Emotion-oriented coping scale involves the use of emotional strategies to reduce stress, where there are emotional responses (get angry, become tense) and in some cases the reaction actually increases stress. The scale of Avoidance-oriented coping describes activities and cognitive changes aimed at avoiding the stressful situation. The range of possible scores of each subscale is 16–80 with higher scores indicating greater use of a given coping style. Cronbach’s alpha coefficient was 0.88 for Task-oriented coping, 0.90 for Emotion-oriented coping, and 0.85 for Avoidance-oriented coping.

#### The 14-Item Resilience Scale

The 14-item Resilience Scale (RS-14) used in this study is a 14-item resilience assessment (Wagnild, 2009) derived from the original Resilience Scale of Wagnild and Young (1993). This questionnaire is largely used in literature. The respondents to RS-14 were asked to state the degree to which they agree or disagree with each item on a 7-point Likert-type scale from 1 (strongly disagree) to 7 (strongly agree). In this research, we adopted the Italian version (Callegari et al., 2016) of this questionnaire (Cronbach’s alpha: 0.89).

#### Intolerance of Uncertainty Scale Short Form

The Italian validation of Intolerance of Uncertainty Scale Short Form (IUS; Lauriola et al., 2016) is composed of 12 items measured on a Likert scale from 1 (not at all agree) to 5 (totally agree). In this questionnaire, uncertainty is conceptualized as a psychological stressor that can threaten an individual’s capacity to cope effectively with situations when there is little or no information. The IUS has two scales: prospective IU and inhibitory IU. The prospective scale measures both the desire for predictability and an individual’s active engagement in seeking information to increase certainty. The inhibitory scale reflects avoidance of uncertainty and paralysis in the face of uncertainty. In this study, Cronbach’s alpha was 0.86 for prospective IU and 0.91 for inhibitory IU.

### Data Analysis

The statistical analyses were conducted using the Statistical Package for Social Science (SPSS) version 25 for Windows (IBM, Armonk, NY, USA). Data were reported as frequencies and percentages for discrete variables and as means and standard deviations for continuous variables. Regarding burnout dimensions, a description of the levels at the MBI scales was reported based upon cutoff scores identified by Sirigatti and Stefanile (1993). Moreover, we conducted a Cluster Analysis, which enables the categorization of participants on the basis of their profiles of responses on a selected set of variables (here, dimensions on the MBI). This approach allows researchers to identify groups that may not emerge *via* classical categorizations (i.e., low, medium, and high) but that nevertheless occur and do have a meaning for participants. The groups identified by the Cluster Analysis were compared on coping styles, intolerance of uncertainty, and resilience through one-way ANOVAs.

In addition, Pearson correlations were performed to explore the association between burnout dimensions and psychological features (coping, resilience, and intolerance of uncertainity). Finally, a set of multiple regression analyses was performed to investigate possible predictors of the burnout dimensions; multiple regression analyses were done separately for each of the three components of burnout as a dependent variable and the variables that were significant from the correlation analysis as predictors.

In all performed analyses, a significance criterion equal to or less than 0.05 was used to determine statistical significance.

## Results

### Descriptive Analysis

The total sample consisted of 102 Italian GPs (64 female). The sociodemographic characteristics and the questionnaire mean scale scores of the participants are presented in Table 1.

**Table 1:** Sociodemographic variables of the sample and descriptive statistics.

**Sociodemographic variables**	**Mean**	**Standard deviation**
**Age**	55.13	11.40
	%	*N*
**Gender**		
Female	62.7	64
Male	36.3	37
Other	1	1
**Years of work experience**		
Less than 3	7.8	8
From 3 to 5	2	2
From 5 to 10	3.9	4
More than 10	86.3	88
**Psychotherapy**		
No	88.2	90
Yes	11.8	12
**Psychological Variables**	**Mean**	**Standard Deviation**
MBI Emotional Exhaustion	26.47	13.33
MBI Depersonalization	7.53	6.13
MBI Personal accomplishment	35.02	6.95
CISS Task-oriented coping	62.38	9.19
CISS Emotion-oriented coping	39.21	12.00
CISS avoidant-oriented coping	45.40	11.02
Resilience	75.85	12.27
IU Prospective	22.12	6.49
IU Inhibitory	10.62	4.91

CISS, coping inventory for stressful situations; IU, intolerance of uncertainty; MBI, Maslach burnout inventory.

Regarding burnout levels, the EE score appears to be the most concerning finding (Table 2); 46.1% of the sample had a high level of EE based on the MBI cutoff (Maslach et al., 1986).

**Table 2:** Levels of burnout in the sample.

	**Low burnout** **Cutoff <17**	**Moderate burnout** **Cutoff 18–29**	**High burnout** **Cutoff >30**
MBI Emotional Exhaustion	30.4%	23.5%	46.1%
	**Low burnout** **Cutoff <5**	**Moderate burnout** **Cutoff 6–12**	**High burnout** **Cutoff >12**
MBI Depersonalization	47.1%	35.3%	17.6%
	**Low burnout** **Cutoff >40**	**Moderate burnout** **Cutoff 36–39**	**High burnout** **Cutoff <36**
MBI Personal accomplishment	28.4%	29.4%	42.2%

MBI, Maslach burnout inventory.

### Hierarchical Cluster Analysis and One-Way ANOVAs

As a first step, a hierarchical Cluster Analysis using Ward’s method was run. We then adopted the squared Euclidean distance to determine profiles of participants according to their *z* scores on each subscale of the MBI (Hair et al., 2009; Berjot et al., 2017). The hierarchical Cluster Analysis suggested a four-cluster solution as shown by an examination of the dendrogram. The Bayesian Index Criterion (Schwarz, 1978) confirmed the four-cluster solution, as the lowest value was observed for this solution. In a second step, to validate the four-cluster solution, a k-mean Cluster Analysis on the numbers of clusters emerging in the hierarchical Cluster Analysis was run (Blashfield and Aldenderfer, 1988; Ransom and Fisher, 1995).

**Figure 1:**
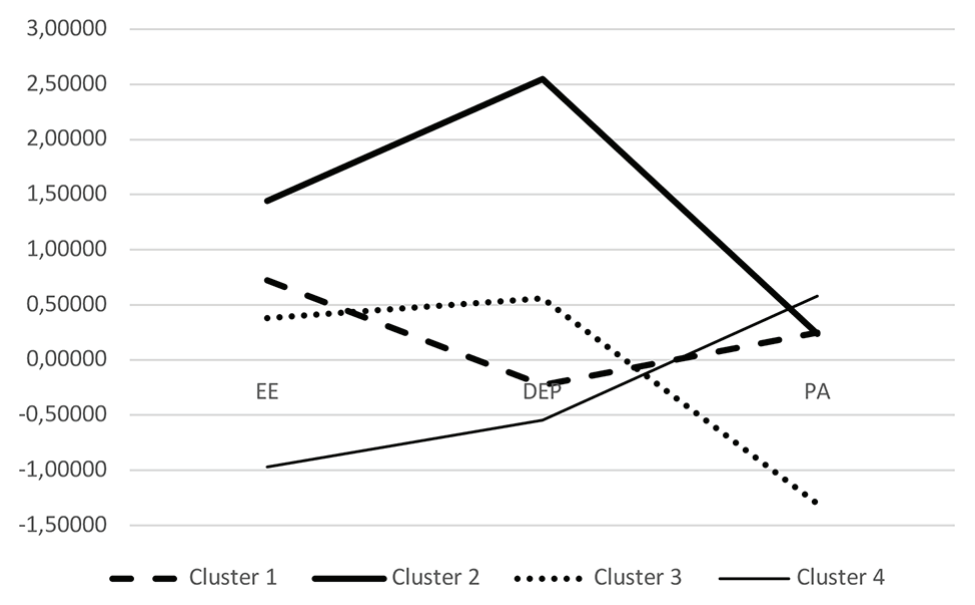
Plot of means for each variable according to clusters. Cluster 1, medium risk burnout; Cluster 2, high burnout; Cluster 3, high risk burnout; Cluster 4, low burnout.

As shown in Figure 1, Cluster 1 (labeled “Medium Risk Burnout” profile, *N* = 30) included GPs who had relatively high levels of emotional exhaustion but medium depersonalization and personal accomplishment. Cluster 2 (“High Burnout” profile, *N* = 6) included GPs who had concomitantly high levels of emotional exhaustion and depersonalization and medium levels of personal accomplishment. Cluster 3 (“High Risk Burnout” profile, *N* = 25) was characterized by moderate levels of emotional exhaustion and depersonalization but also very low levels of personal accomplishment. Finally, Cluster 4 (“Low Burnout” profile, *N* = 41) was characterized by low levels of emotional exhaustion and depersonalization and a moderate level of personal accomplishment.

Means and SD for each dimension of the MBI scale according to the clusters were reported in Table 3.

**Table 3:** Mean scores and standard deviations for each dimension of the MBI scale according to clusters.

	*N*	Mean	SD
**Emotional exhaustion**			
Medium Risk Burnout	30	0.72	0.57
High Burnout	6	1.44	0.60
High Risk of Burnout	25	0.38	0.62
Low Burnout	41	−0.97	0.51
**Depersonalization**			
Medium Risk Burnout	30	−0.23	0.62
High Burnout	6	2.55	0.78
High Risk of Burnout	25	0.56	0.71
Low Burnout	41	−0.55	0.60
**Personal accomplishment**			
Medium Risk Burnout	30	0.25	0.62
High Burnout	6	0.24	0.92
High Risk of Burnout	25	−1.30	0.48
Low Burnout	41	0.58	0.73

Finally, we ran a series of one-way ANOVAs with clusters as an independent variable and each dimension – CISS, IU, and Resilience – as a dependent variable. As shown in Table 4, significant differences emerged on CISS Task-oriented (*F* = 9.49, *p* = 0.00) and Emotion-Oriented (*F* = 16.78, *p* = 0.00). Specifically, Bonferroni *post hoc* analysis showed lower levels of CISS Task-oriented coping in High Risk GPs compared to both Medium Risk (*p* = 0.05) and Low Burnout GPs (*p* = 0.05); higher levels of CISS Emotion-Oriented in High Burnout GPs compared to all the other groups (always *p* = 0.05). Regarding Resilience, higher levels emerged in Medium Risk GPs than in High Risk GPs (*p* = 0.05) and in Low Burnout than in High Risk (*p* = 0.05). Finally, higher levels of IU Prospective emerged in High Risk GPs than in Low Burnout (*p* = 0.05), and higher levels of IU Inhibitory were found in High Burnout GPs compared to both Medium Risk and Low Burnout GPs (always *p* = 0.05).

**Table 4:** One-way ANOVAs between cluster profiles on coping styles, resilience, and intolerance of uncertainty.

	Medium Risk Burnout		High Burnout		High Risk Burnout		Low Burnout			
	Mean	SD	Mean	SD	Mean	SD	Mean	SD	F	p
CISS Task-oriented	63.03	8.31	61.67	11.78	55.28	9.10	66.34	6.94	9.49	0.00
CISS Emotion-oriented	38.47	9.44	58.83	14.62	45.60	8.05	32.98	10.51	16.78	0.00
CISS Avoidant-oriented	43.40	10.74	56.17	13.89	44.52	9.98	45.83	10.88	2.41	0.07
Resilience	77.43	10.16	74.83	18.08	65.00	12.77	81.46	7.68	12.94	0.00
IU Prospective	22.33	6.94	25.83	6.77	24.48	5.21	19.98	6.28	3.49	0.02
IU Inhibitory	10.57	5.10	17.00	5.83	11.76	4.54	9.02	4.09	5.96	0.00

CISS, coping inventory for stressful situations; IU, intolerance of uncertainty.

### Correlational Analysis

Pearson correlational analyses were carried out to explore relationships between burnout subscales and sociodemographic characteristics. A correlation between MBI Depersonalization and age (*r* = −0.300, *p* = 0.002) and years of work experience (*r* = −0.283, *p* = 0.004) emerged, whereas no significant relationships were found for the gender and burnout dimensions.

Regarding the relationships between burnout and coping dimensions (Table 5), Pearson correlation analysis showed that MBI Emotional Exhaustion scale was positively correlated with CISS Emotion-oriented (*r* = 0.495, *p* = 0.001) and negatively with CISS Task-oriented (*r* = −0.247, *p* = 0.012); MBI Depersonalization scale correlated positively with CISS Emotion-Oriented (*r* = 0.522, *p* = 0.001) and Avoidance-oriented (*r* = 0.233, *p* = 0.019) and negatively with CISS Task-oriented (*r* = −0.221, *p* = 0.025); MBI Personal Accomplishment scale was correlated negatively with CISS Emotion-oriented (*r* = −0.312, *p* = 0.001) and positively with CISS Task-oriented (*r* = 0.590, *p* = 0.001).

**Table 5:** Correlation between burnout dimension and psychological features.

	CISS Emotional	CISS Task	CISS Avoidance	Resilience	IU Prospective	IU Inhibitory
MBI Emotional Exhaustion	0.495^**^	−0.247^*^	0.041	−0.247^*^	0.279^**^	0.305^**^
MBI Depersonalization	0.522^**^	−0.221^*^	0.233^*^	−0.200^*^	0.232^*^	0.192
MBI Personal accomplishment	−0.312^**^	0.590^**^	0.136	0.686^**^	−0.267^**^	−0.265^**^

MBI, Maslach burnout inventory; CISS, coping inventory for stressful situations; Task, task-oriented coping; Emotional, emotion-oriented coping; Avoidance, avoidance-oriented coping; IU, intolerance of uncertainty.

* *p* < 0.05;

** *p* < 0.001.

Regarding the Resilience scale (Table 5), the analysis highlighted a significant positive correlation with the MBI Personal Accomplishment score (*r* = 0.686, *p* = 0.001) and a negative correlation with MBI Emotional Exhaustion (*r* = −0.247, *p* = 0.012) and Depersonalization (*r* = −0.200, *p* = 0.044).

Finally, the relationships between Burnout dimensions and Intolerance of Uncertainty (IU) subscales were explored (Table 5). MBI Emotional Exhaustion was correlated with IU Prospective and Inhibitory (respectively, *r* = 0.279, *p* = 0.005; *r* = 0.305, *p* = 0.002); MBI Depersonalization was positively correlated with IU Prospective (*r* = 0.232, *p* = 0.019); MBI Personal Accomplishment, on the other hand, was negatively correlated with IU Prospective and IU Inhibitory (respectively, *r* = −0.267, *p* = 0.007; *r* = −0.265, *p* = 0.007).

### Regression Analysis

Since significant correlations between each dimension of burnout and participants’ psychological and sociodemographic features emerged, three multiple linear regression models were performed to investigate possible predictors of MBI Emotional Exhaustion, Depersonalization, and Personal Accomplishment measures.

The first model of linear regression with MBI Emotional Exhaustion as the dependent variable and Resilience, CISS Emotion-oriented and Task-oriented, and IU Prospective as predictors was significant. The model predicted 27% of BMI Emotional exhaustion (*R*^2^ = 0.274; adjusted *R*^2^ = 0.244; *p* < 0.001) with only CISS Emotion-oriented scores found to be a significant predictor (beta = 0.461; *p* < 0.001).

A linear regression analysis having MBI Depersonalization as the dependent variable and age, resilience, all dimensions of CISS (Task-oriented, Emotion-oriented, and Avoidance-oriented), and IU Prospective as predictors was run. This model was significant and predicted 36% of MBI Depersonalization scores (*R*^2^ = 0.365; adjusted *R*^2^ = 0.318; *p* < 0.001); age and CISS Task-oriented and Emotion-oriented emerged as significant predictors (respectively, beta = 0.183, *p* = 0.034; beta = −0.298, *p* = 0.023; beta = 0.496, *p* < 0.001).

The last model of linear regression with MBI Personal Accomplishment as a dependent variable and Resilience, IU Inhibitory, and CISS Task-oriented and Emotion-oriented as predictors was run. This model was significant and predicted 51% of MBI Personal Accomplishment (*R*^2^ = 0.512; adjusted *R*^2^ = 0.486; *p* < 0.001) and showed that only Resilience was a predictor of MBI Personal Accomplishment (beta = 0.500; *p* < 0.001).

## Discussion

This research explored the relationships among psychological phenomena (coping, resilience, and perception of uncertainty) and Burnout among GPs in Italy. The extraordinary impact of the COVID-19 emergency on GPs, as frontline medical providers, was in part produced by the uncertainty of the procedures and treatments required and the immediate saturation of hospitals for critical case management. GPs had to respond directly to a huge number of requests without clear prevention or screening instruments. All these aspects affected the GPs, who, according to the MBI cutoff, simultaneously showed high perception of competence and productivity (the 28.4% of the sample had a high level of Personal Accomplishment at the MBI) and a reduction in emotional resources (the 46.1% had a high level of Emotional Exhaustion). In addition to a classification of participants according to existing cutoff scores, we utilized an alternative technique, cluster analysis, which provides criteria specific to the population under study. This choice allowed us to rise above “all or nothing” conceptualizations (i.e., people suffer from burnout or they do not) and to identify subgroups of burnout according to the individual experience of work (Berjot et al., 2017). It also allowed for the identification of specific groups or at-risk groups, which may enable the selection and the deployment of specific prevention and intervention programs (Clatworthy et al., 2005). The cluster analysis showed four different profiles, labeled “Low Burnout,” “High Burnout,” “Medium Risk Burnout,” and “High Risk Burnout.”

Results partially confirmed the cutoff categorization, showing 40% of the sample in the Low Burnout profile and only about 5% in the High Burnout profile.

Cluster analysis allows for a more qualitative reading using burnout scales. It highlighted two risk profiles: a “Medium Risk Burnout” cluster (30% of the sample) and a “High Risk of Burnout” cluster (25% of the sample). Those two groups cannot be classified as suffering burnout, but they emerged by cluster analysis as groups that can be described as being “at risk of burnout,” composed of professionals who may 1 day suffer burnout if environmental demands and threats remain high while resources remain low. Specifically, the “Medium Risk Burnout” profile included GPs who had relatively high levels of emotional exhaustion but medium depersonalization and personal accomplishment while the “High Risk Burnout” profile was characterized by moderate levels of emotional exhaustion and depersonalization but very low levels of personal accomplishment. In this last case, the gratification that work can offer cannot act as a personal resource, protecting against the risk of depersonalization and emotional exhaustion. Comparing all four groups strengthens this observation, highlighting that the high risk group showed lower resilience and did less task-oriented coping than the medium risk group and demonstrated more need for control than the low burnout group. These specific characteristics can be used as indications for differentiated interventions in support of GPs, focusing and intervening on specific pandemic reaction patterns. Moreover, the group with high burnout was characterized by higher use of emotional strategies to reduce stress than the other three groups and higher avoidance of uncertainty, as well as paralysis in the face of it.

Starting from these first analyses, and from the correlations’ results, the regressions were performed in order to examine which psychological features predicted burnout levels. Results showed, according to the previous comparison between burnout profiles, that depletion of the emotional resources was related to emotion-oriented coping, so the activation of emotional strategies was associated with a less functional response to the emergency. These data are probably affected by the fact that GPs’ activities were limited by lockdown rules and the impossibility of using concrete clinical findings to manage patients’ symptoms and disease progression, relying instead on patient reports of their subjective experiences. Emotion-oriented coping is strictly related to a higher sense of responsibility to solve other problems (i.e., I blame myself for not knowing what to do) and take care of the situation, so the missing doctor-patient relationship and the absence of medical protocols generated a higher sense of inefficacy and frustration in the immediate reactions to the pandemic. Moreover, it is possible that GPs did not have the resources to experience and process the intense emotional reactivity linked to the pandemic, and to the perception of the risk of being infected, at least in the immediate emergency. This may have left many of them with intense, unregulated emotions, which could interfere with professional response.

This hypothesis is supported by the results related to the Depersonalization scale of the MBI that was predicted by high levels of Emotion-oriented coping and low levels of Task-oriented coping. The primary resource to avoid the tendency of viewing coworkers and clients as dehumanized objects seemed to be the task-oriented coping that, consistent with previous research, represented a proactive and concrete response to stress (Chang and Chan, 2015; Lall et al., 2019). In a highly stressful situation like the COVID-19 emergency, emphasizing a task-oriented action, planning, and problem-solving, rather than an emotion-oriented strategy, appears to be a more effective way to provide care without depersonalization. It is also important to note that depersonalization was the only variable related to age and years of work experience; this finding is supported by the literature (Lim et al., 2010), where a longer period of exposure to suffering tends to generate more depersonalization. The years of work variable was found to have the most significant positive correlations to Burnout (Iglesias et al., 2010). This finding is important to take into consideration in understanding any GP turnover that may follow this traumatic situation, as well as in simply understanding the impact of the pandemic on GPs. On the basis of these results, it would be appropriate for medical systems in Italy and beyond to develop programs for preventing and treating burnout syndromes in GPs.

The findings in this paper contribute to our field’s understanding of the benefits and drawbacks of coping strategies focused on emotions or on problem-solving, which appear to be dependent on context. Understanding that in the context of a crisis like a pandemic, problem-solving strategies may do more to prevent burnout and depersonalization among medical professionals and can help to tailor training and preparation for these frontline providers in the future.

Furthermore, it is clear from these results that Resilience has an important role: it is a significant predictor of burnout Personal Accomplishment, according to the literature (Taku, 2014; Kutluturkan et al., 2016). Resilience is a person’s ability to manage his or her sense of responsibility in an unfamiliar and chaotic situation like the COVID-19 pandemic and can have a meaningful impact on his or her capacity to work effectively. In fact, resilience – defined as a person’s capacity for or produced outcome of successful adaptation despite challenging or threatening circumstances (Masten et al., 1990) – is positively correlated with feelings of competence, productivity, and success. Moreover, our findings showed that the High Risk Burnout group had lower scores in Resilience than all other groups, suggesting that this feature is important to prevent burnout. The fact that GPs’ capacity for resilience in the present pandemic situation is connected in this research with their sense of work efficacy suggests that resilience may be an important part of professional identity in the medical field, as may be the case for a general belief in medical practice, even when immediate solutions and pharmacological cures fall short.

Finally, the perception of the COVID-19 pandemic as an unpredictable situation was analyzed using an assessment scale (Intolerance of Uncertainty Scale) that revealed two factors as principal reactions to uncertainty: the desire for predictability and uncertainty paralysis (Hong and Lee, 2015). Although the scales were not significant predictors of burnout in the regression analyses, they were positively correlated with emotional exhaustion and negatively correlated with personal accomplishment. The unpredictable situation and unfamiliar scenarios had a strong impact on emotional distress and raised psychological defenses. We can speculate that chaotic situations and constantly changing protocols affected self-efficacy and made a direct impact on GPs’ personal and emotional lives.

There are several limitations inherent in the present study. First, since the COVID-19 pandemic affected regions of Italy in different ways, it would be interesting to have a larger sample to be able to verify whether the relationships between burnout and psychological characteristics are different depending on the severity of the health emergency in any given region. A second limitation involves the absence of a control group, which would be useful in future investigations for performing comparative analysis. Hospital staff, rather than other emergency management personnel (such as the army force), could represent a comparison group. This would allow for the identification of specific stress reaction patterns in the different groups. In addition, long-term follow-up to collect further data on GPs’ health status would help to verify the predictive role of burnout on the long-term psycho-physical health of participants.

In conclusion, the results of this study showed an impact on GPs’ work management during the COVID-19 emergency. Implementing task-oriented problem management, rather than emotional strategies, appears to protect against burnout. It is possible that the emotions related to the pandemic are too intense to be regulated and used in order to manage the professional issues that the COVID-19 pandemic involves. Moreover, these results support the need to organize both training and psychological interventions for GPs, with the aim of providing them with greater skills in emotional regulation in general and, over the course of an emergency, supporting their capacity to process intense emotional experiences, which can impact the quality of medical work.

## Data Availability Statement

The raw data supporting the conclusions of this article will be made available by the authors, without undue reservation.

## Ethics Statement

The studies involving human participants were reviewed and approved by Ethics Committee of Department of Dynamic and Clinical Psychology, University of Rome, Sapienza. The patients/participants provided their written informed consent to participate in this study.

## Author Contributions

CDM contributed to all the phases of the study. SM participated in research design development, in results interpretation, and in writing and editing the manuscript. RM participated in results interpretation and in writing the manuscript. MDT participated in research design. All authors contributed to the article and approved the submitted version.

### Conflict of Interest

The authors declare that the research was conducted in the absence of any commercial or financial relationships that could be construed as a potential conflict of interest.
